# Dispersal of *Rhagoletis cerasi* in Commercial Cherry Orchards: Efficacy of Soil Covering Nets for Cherry Fruit Fly Control

**DOI:** 10.3390/insects4010168

**Published:** 2013-03-12

**Authors:** Claudia Daniel, Brian Baker

**Affiliations:** Research Institute of Organic Agriculture (FiBL), Ackerstrasse 21, Postfach 219, CH-5070 Frick, Switzerland; E-Mail: bpb33@cornell.edu

**Keywords:** Diptera, Tephritidae, Migration, netting, organic agriculture

## Abstract

Demand for organic cherries offers producers a premium price to improve their commercial viability. Organic standards require that producers find alternatives to pesticides. Soil treatments to control the European cherry fruit fly *Rhagoletis cerasi* (L.) (Diptera: Tephrididae) appear to be an attractive option. However, soil treatments can only be effective if the migration of flies is low, because mature flies may migrate from near-by trees for oviposition. To examine the general potential of soil treatments and to understand the dispersal and flight behaviour of *R. cerasi* within orchards, experiments using netting to cover the soil were conducted in two orchards with different pest pressure during two years. The netting reduced flight activity by 77% and fruit infestation by 91%. The data showed that the flies have a dispersal of less than 5 m within orchards, which is very low. The low thresholds for tolerance for infested fruit in the fresh market creates a strong economic incentive for control, therefore, soil covering is a promising strategy for controlling *R. cerasi* in commercial orchards.

## 1. Introduction

The European cherry fruit fly, *Rhagoletis cerasi* (L.) (Diptera: Tephritidae) is a highly destructive pest of sweet cherries in Europe [1]. The adult flies emerge from the soil in May and June and begin to lay eggs about ten days after emergence. The larvae develop inside the cherries. At the third instar, the larvae leave the fruit, drop to the soil and within hours start to pupate under the tree canopy. *R. cerasi* is univoltine and overwinters as pupae [2,3]. Without insecticide treatment 100% of the fruit can be infested [4]. *R. cerasi* poses a challenge to cherry growers because of the low tolerance level of the fresh market to damaged fruit—with a maximum of two per cent of infested fruits. The tolerance level of six percent in cherries for canning industry is also often exceeded. Because the infested fruit cannot be sorted out, the entire crop is rejected if tolerance levels are not met. The disqualification of fresh market cherries or cannery cherries to distillery quality considerably reduces the market price, which causes serious financial losses. *R. cerasi* is the only pest insect that requires treatment of cherry fruit in Europe. All other insect pests of cherries (*Operophtera brumata* Linnaeus, *Argyresthia pruniella* Clerck, *Myzus cerasi* Fabricius) can be easily controlled in the early spring by pre-bloom applications. A control method for *R. cerasi* by soil applications seems attractive in terms of producing insecticide residue-free cherries. Because *R. cerasi* pupae spend more than 10 months per year in the soil [2] and because the area of pupation is strictly limited to the surface directly under the canopy of infested trees [3], the possibility of soil treatments is appealing [5].

Soil treatments have been tested for their efficacy for over 100 years [6]. Methods such as soil cultivation or compression, and chemical treatment with arsenic compounds or kerosene all were either labour intensive [6,7], ineffective [8] or showed unacceptable side effects [9]. When organo-chlorine insecticides such as DDT became available in the 1950s [10], the research on soil treatments was abandoned. With the ban of Dimethoate and growing knowledge on microbial biological control agents of *R. cerasi*, soil treatments are again being considered. Laboratory experiments demonstrated an efficacy of entomopathogenic nematodes [11,12] or fungi [13,14] on *R. cerasi*.

However, soil treatments can be effective only if fly migration is low, because mature flies would migrate from near-by trees. Thiem [8] hypothesized that most of the flies migrate from near-by *Lonicera* plants to the cherry trees. However, dispersal of flies by wind was considered to be more important than active flight ability [15]. According to Wiesmann [9], flies rarely migrate more than 300 m from their emergence sites and migration is induced only in cases of insufficient supply of host fruit or after harvest. This observation is supported by Katsoyannos *et al.* [16], who stated that flies move from early ripening varieties to later ripening varieties and from there on to *Lonicera*, driven by oviposition pressure of females. Detailed experiments on dispersal behaviour of *R. cerasi* were conducted within the framework of the ‘sterile-males-technique’. Flight studies in the laboratory showed that flies are capable of flying more than one kilometre in 24 hours [17]. Under field conditions, however, 82% of marked and released flies were recaptured at a distance of less than 100 m, only 0.7% of the flies were recaptured at a distance of 500 m and none at a distance of 600 m [18]. Flights over long distances were only observed across open fields with direct visibility of the next tree [19]. However, all studies cited above were conducted in landscapes with some scattered, standard trees. No data are available on dispersal and migration behaviour of *R. cerasi* within orchards.

In order to examine the general potential of soil treatments and to understand the dispersal and flight behaviour of *R. cerasi* within orchards, experiments using netting to cover the soil were conducted in two orchards.

## 2. Materials and Methods

**Experimental orchards:** The experiments were conducted in two commercial, organically managed orchards (orchard A and B) in northwestern Switzerland in 2005 and 2006. The orchard A consisted of 82 cherry trees of different varieties. Trees were five metres tall and yielded an average of 15 kg cherries each. The orchard A was arranged in five rows (replicates) with 20 trees per row, a row length of 100 m and a distance of 5 m between the trees. The orchard B consisted of 25 cherry trees of different varieties. Trees were five metres tall and yielded 7.5 kg cherries each. The orchard B was arranged in three rows (two replicates per row) with a row length of 65 to 90 m and a distance of 8 m between the trees. An inspection of infestation rates in both orchards during the preceding years indicated that the distribution of *R. cerasi* was homogenous within the orchards, with a tendency toward slightly higher infestation rates in the centre of the orchard. In the year before the experiments, an average of 29.25 flies per trap and per tree were captured during the whole flight period in orchard A (2004). In orchard B (2005), 106.75 flies per trap and per tree were captured during the whole flight period in the year before the experiment.

**Figure 1:**
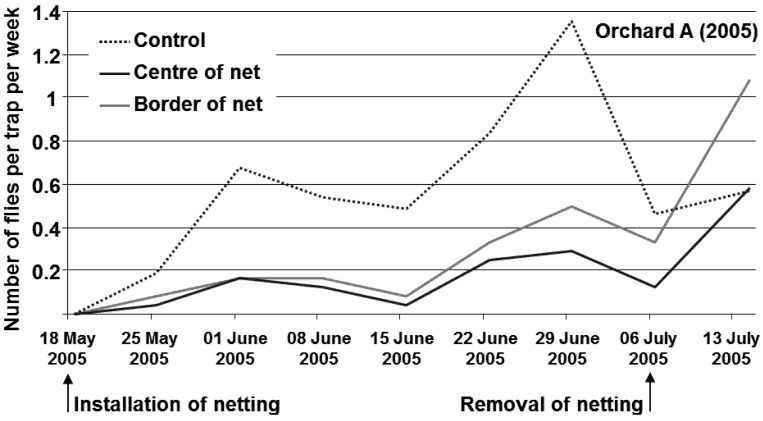
Flight activity of *R. cerasi* in orchard A in 2005.

**Figure 2:**
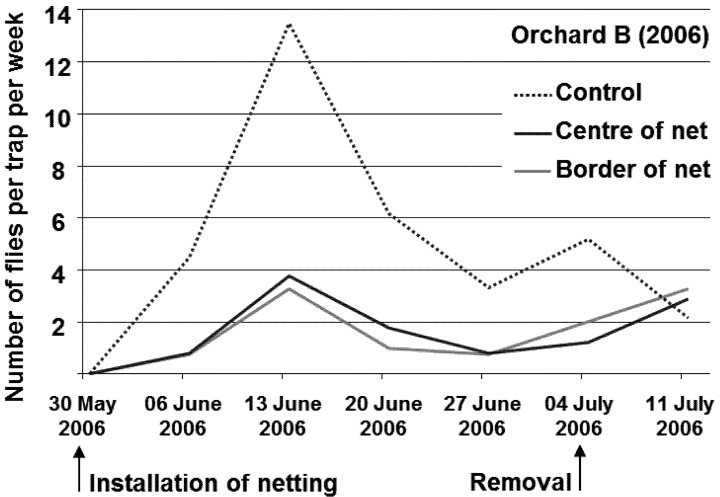
Flight activity of *R. cerasi* in orchard B in 2006.

**Installation of soil covering nets:** In the middle of each row, 40 (orchard B) to 50 (orchard A) running metres of the soil under the tree canopies were covered with a fine mesh netting (BiocontrolNet, Andermatt Biocontrol AG, Grossdietwil, Switzerland, white colour, 0.8 mm mesh width; 50 m long, 3 m wide) before the beginning of the emergence period. On each side of the rows, 12 (orchard B) and 25 running metres (orchard A) were left uncovered to provide an untreated control. The edges of the netting were buried to prevent flies from escaping. However, not all holes could be closed completely around the tree trunks. The soil under all other cherry trees in a perimeter of 50 m around the experimental orchards was covered in a similar manner. Dates of installation and removal of nettings are indicated in Figure 1, Figure 2.

**Monitoring of flight activity:** Fly activity was monitored using one yellow sticky trap (Rebell® amarillo, Andermatt Biocontrol AG, Grossdietwil, Switzerland) per tree. Flies per trap were counted at weekly intervals. Traps remained on the trees until one week after removal of the netting to examine whether flies were able to survive under the net cover.

**Fruit infestation:** The fruit infestation could not be evaluated in orchard A because the trees yielded no fruit due to a detrimental attack of *O. brumata* in early spring. In orchard B, a sample of 50 cherries per tree was taken according to the harvesting time of the different varieties between 17 June and 04 July 2006. The cherries were dissected under the binocular microscope to determine the exact infestation level of *R. cerasi* with eggs, larval instars and damaged fruit, abandoned by larvae going into pupation. 

**Statistical analysis**: JMP version 5.0.1.2. was used for all statistical analyses. Normality of data and homogeneity of variance were tested before performing a one-way ANOVA [treatment; Data from orchard A] or a two-way ANOVA [treatment, cherry variety; data from orchard B]. Means were compared by Tukey HSD post hoc tests (α = 0.05). Data are presented in the figures and the text as means with standard errors. With respect to the flight activity and fruit infestation, the following treatments were compared: (1) control, without covering; (2) border area of netting (less than 10 m distance to the border of the netting); (3) centre of netting.

**Economic analysis:** An economic analysis was conducted in order to estimate the economic feasibility of soil covering nets. The analysis was based on Swiss prices. The conversion rate for CHF to € was assumed to be 1.20. No discount rate or depreciation schedule was used. Material costs for netting was €1.13/m^2^ (Prize for Biocontrol Net 0.8 sold by Andermatt Biocontrol AG, Switzerland). Netting is expected to have a useful life span of eight years based on performance in vegetable production. Because it is not necessary to cover the whole surface, but only the area directly under the tree canopy the area covered per ha is reduced to 7,500 m^2^. Labour input of 120 h/ha was estimated from the time needed to set-up the experiments. Labour costs of €17.50 per hour were used according to the Arbokost model [20], a business management simulation program for cherry production based on data evaluated in Switzerland. Analysis was conducted for two model orchards based on data from the Arbokost model: Orchard 1: intensive dwarf rootstock orchard, cherries for fresh consumption, 800 trees per hectare, yield: 14 kg/tree. Orchard 2: semi-intensive production, cherries for canning industry, 300 trees per hectare, yield: 25 kg/tree. An additional worst-case-scenario (Orchard 3) was calculated for an orchard near the end of its productive life with many trees removed, a density of 200 trees/ha and an expected yield of 7.5 kg per tree. Swiss market data for average prices of organic distillery (€1.00/kg), organic cannery (€3.33/kg) and organic fresh market (€6.50/kg) prices from 2009–2012 were used for the calculations. For the worst case scenario we used ten year market low prices from 2002–2005 (organic distillery quality: €0.92/kg, organic cannery quality: €2.38/kg; organic fresh market quality: €4.58/kg) and we assumed a reduced life span of netting material of only 4 years.

## 3. Results

**Flight activity:** Figure 1, Figure 2 show the pattern of flight activity in orchard A and B, respectively. The flight period in orchard A started shortly after installing the netting and reached a peak during the warm and sunny period from 14 June to 28 June 2005. Over the whole flight period only 4.5 flies per trap per tree were caught in the control plots. A higher flight activity was observed in orchard B in 2006. Flight activity was seven times higher than in the orchard A in 2005: over the whole flight period, 32.4 flies per trap were caught in the control plots. The flight period in orchard B started shortly after installing the netting and reached a peak during the warm, sunny period from 7 June to 14 June 2006. Flight activity was mainly influenced by climatic conditions. Graphs of the different treatments showed similar patterns of activity. No change in activity was found toward the end of the flight period. After the netting was removed, however, the captures in the treated plots increased. 

**Figure 3:**
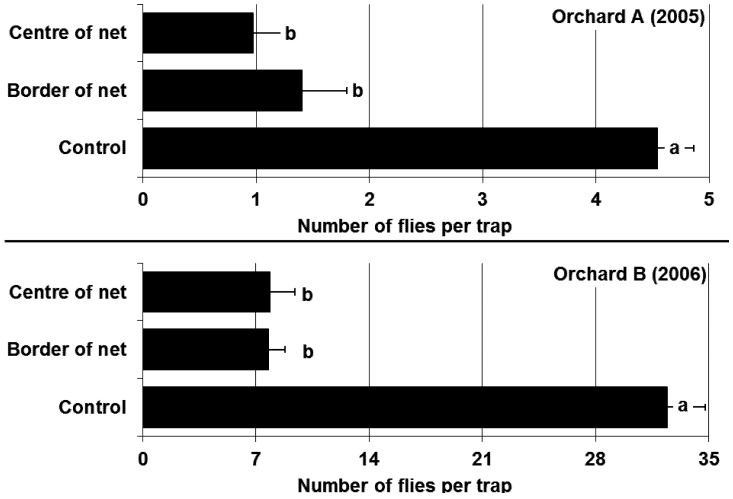
Effects of soil covering with netting on average number of *R. cerasi* (±se) on yellow sticky traps in orchard A (2005) and orchard B (2006). Statistical analysis: orchard A: one-way ANOVA: F_2,12_ = 34.99, p < 0.001; Orchard B: two-way ANOVA [treatment, cherry variety]: treatment: F_2,9_ = 118.46, p < 0.001, variety: F_4,9_ = 4.70, p = 0.03; Tukey HSD-Test α = 0.05; different letters show significant differences.

Cumulative captures of flies per trap during the whole coverage period are given in Figure 3. The netting significantly reduced the number of flies and showed an efficacy of 76 to 77% using Abbott’s formula [21]. No significant differences were found between the border within 10 m of the control and the centre of the netting. Differences in numbers of flies per trap after removal of the netting were not significant (statistical analysis orchard A: one-way ANOVA: F_2,12_ = 2.49, p = 0.12; orchard B: two-way ANOVA [treatment, cherry variety]: treatment: F_2,9_ = 0.73, p = 0.51, variety: F_4,9_ = 0.91, p = 0.50).

**Figure 4:**
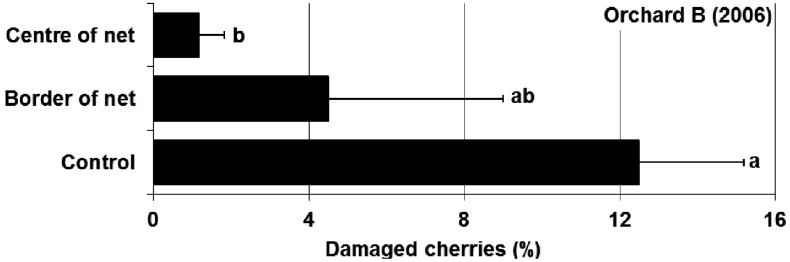
Effects of soil covering with netting on percentage infestation rate of cherries with *R. cerasi* larvae (±se) in the orchard B (2006). Statistical analysis: two-way ANOVA [treatment, cherry variety]: treatment: F_2,9_ = 6.61, p = 0.02, variety: F_4,9_ = 1.36, p = 0.32; Tukey HSD-Test α = 0.05; different letters show significant differences.

**Fruit infestation: **Infestation level at harvest was significantly reduced by soil netting (Figure 4). In the control plots, 12.5% of the cherries were damaged by *R. cerasi* larvae. With an infestation level of 4.5%, an efficacy of 64% was achieved for the trees at the border of the netting. In the centre of the netting 1.2% of fruit were infested, indicating an efficacy of 91% (Abbott’s formula).

**Economic analysis:** The installation of the netting caused annual labour costs of €2’100 per hectare. Material costs for netting with a life span of eight years were €1’063 per hectare per year. Thus, total netting costs sum up to €3’162 per hectare year (Table 1). With a reduced life span of netting of only four years the costs would increase to €4’225 per year (see worst case scenario in Table 1). Based on the experimental data, orchards that are untreated with pesticides and not covered with netting will exceed tolerance levels for *R. cerasi* infestation. The results show, that netting will almost certainly be able to reduce infestation levels to cannery grade and have a high probability of making fresh grade. Table 1 presents the gross return on the installation of netting in organic orchards. The returns on installing the netting were positive in all scenarios except the worst case scenario.

**Table 1:** Gross return (€) on the installation of netting in Swiss organic cherry orchards (see materials and methods for assumptions used for the calculations).

Return	Model Orchard 1Fresh Market Quality	Model Orchard 2Canning Quality	Model Orchard 3Worst Case Scenario
Yield (kg/ha)	11’200	7’500	1’500
Revenue without Netting (€)	11'200	7'500	1'375
Revenue with Netting (€)	72'800	25'000	3'563
Netting Costs (€)	3'163	3’163	4'225
Gross Margin (€)	58'438	14'338	−2'038

## 4. Discussion

Covering the soil under the tree canopies reduced the flight activity of *R. cerasi* by 76–77%. This effect was also observed at the border of the netting in the immediate vicinity of the control trees without soil covering, indicating that flies do not move more than 5 m within an orchard. The results were obtained from two experiments in two years and in different locations with different flight intensities of *R. cerasi*. The number of flies per trap was mainly influenced by weather conditions, with peak activity observed during sunny and warm periods. No change in activity was found toward the end of the flight period in either experiment, indicating that mature females did not show an increased migration. Whether the few flies found on the traps over covered soil escaped through holes around the tree trunks or migrated from neighbouring trees could not be determined. Cherry fruit fly dispersal within a compact orchard is therefore considered to be low. Throughout the whole flight period, a large number of flies seemed to remain in the tree under which they emerged. This observation is unusual, as different authors [9,22] have shown an increased migration in the event of fruit shortage or after harvest. However, migration did not increase within the orchard with either the total lack of fruit in Orchard A or the early harvest of the variety Magda (17 June 2006) in Orchard B. However, the captures in the treated plots increased immediately after removal of netting, which suggests that flies survived under the netting. Although the effect of visual interference on fly behaviour is unlikely, it cannot be ruled out given the applied experimental design. Flies might have been deterred from landing on trees above covered soil by reflectance of light off the netting or other visual cues. After removal of the netting, flies from adjacent trees might have moved onto the treatment trees because the visual interference of the netting disappeared.

Fruit infestation in the centre of the netting (1.2%) was reduced to a level below the 2% economic threshold for fresh market quality. Therefore, soil netting is promising strategy for controlling *R. cerasi* in orchards. The combination of this strategy with perimeter trapping of immigrating flies using sticky traps and baits [23], might even improve the efficacy of soil netting of entire orchards. The economic analysis showed that installation of netting appears to be an economically viable strategy for intensively and semi-intensively managed organic systems. However, gross margins do not reflect any costs of production other than the netting, so it is not possible to determine whether any of the scenarios are profitable. Nevertheless, it is possible to determine whether, all other things being equal, installation of netting yields a positive return. The returns on installing the netting were positive in all scenarios except the worst case scenario.

## 5. Conclusions

Soil netting offers a promising strategy to control the European cherry fruit fly. Fruit infestation was reduced to a level below the economic threshold for fresh market quality. The economic analysis showed that installation of netting is economically viable. Moreover, considering the good laboratory results with entomopathogenic fungi [13], the development of a field application strategy for soil treatments with entomopathogenic fungi could be promising.
